# SOFA and mortality endpoints in randomized controlled trials: a systematic review and meta-regression analysis

**DOI:** 10.1186/s13054-017-1609-1

**Published:** 2017-02-24

**Authors:** Harm-Jan de Grooth, Irma L. Geenen, Armand R. Girbes, Jean-Louis Vincent, Jean-Jacques Parienti, Heleen M. Oudemans-van Straaten

**Affiliations:** 1 Department of Intensive Care, VU University Medical Center, De Boelelaan 1117, 1081 HV Amsterdam, The Netherlands; 2 Department of Intensive Care, Erasme Hospital, Université Libre de Bruxelles, Brussels, Belgium; 3 Unité de Biostatistique et de Recherche Clinique, Centre Hospitalier Universitaire de Caen, Caen, France; 4 EA4655 « Risques microbiens », Faculté de Médecine, Université de Caen Normandie, Caen, France

**Keywords:** Critical care trials, Multiple organ failure, Sepsis, Surrogate endpoints

## Abstract

**Background:**

The sequential organ failure assessment score (SOFA) is increasingly used as an endpoint in intensive care randomized controlled trials (RCTs). Although serially measured SOFA is independently associated with mortality in observational cohorts, the association between treatment effects on SOFA vs. effects on mortality has not yet been quantified in RCTs. The aim of this study was to quantify the relationship between SOFA and mortality in RCTs and to identify which SOFA derivative best reflects between-group mortality differences.

**Methods:**

The review protocol was prospectively registered (Prospero CRD42016034014). We performed a literature search (up to May 1, 2016) for RCTs reporting both SOFA and mortality, and analyzed between-group differences in these outcomes. Treatment effects on SOFA and mortality were calculated as the between-group SOFA standardized difference and log odds ratio (OR), respectively. We used random-effects meta-regression to (1) quantify the linear relationship between RCT treatment effects on mortality (logOR) and SOFA (i.e. responsiveness) and (2) quantify residual heterogeneity (i.e. consistency, expressed as *I* ^2^).

**Results:**

Of 110 eligible RCTs, 87 qualified for analysis. Using all RCTs, SOFA was significantly associated with mortality (slope = 0.49 (95% CI 0.17; 0.82), *p* = 0.006, *I* ^2^ = 5%); the overall mortality effect explained by SOFA score (*R* ^2^) was 9%. Fifty-eight RCTs used Fixed-day SOFA as an endpoint (i.e. the score on a fixed day after randomization), 25 studies used Delta SOFA as an endpoint (i.e. the trajectory from baseline score) and 15 studies used other SOFA derivatives as an endpoint. Fixed-day SOFA was not significantly associated with mortality (slope = 0.35 (95% CI −0.04; 0.75), *p* = 0.08, *I* ^2^ = 12%) and explained 3% of the overall mortality effect (*R* ^2^). Delta SOFA was significantly associated with mortality (slope = 0.70 (95% CI 0.26; 1.14), *p* = 0.004, *I* ^2^ = 0%) and explained 32% of the overall mortality effect (*R* ^2^).

**Conclusions:**

Treatment effects on Delta SOFA appear to be reliably and consistently associated with mortality in RCTs. Fixed-day SOFA was the most frequently reported outcome among the reviewed RCTs, but was not significantly associated with mortality. Based on this study, we recommend using Delta SOFA rather than Fixed-day SOFA as an endpoint in future RCTs.

**Electronic supplementary material:**

The online version of this article (doi:10.1186/s13054-017-1609-1) contains supplementary material, which is available to authorized users.

## Background

The sequential organ failure assessment (SOFA) score was developed by an international group of experts to describe the time course of multiple organ dysfunction using a limited number of routinely measured variables [1]. The function of six organ systems is scored from 0 (no organ dysfunction) to 4 (severe organ dysfunction), and the individual organ scores are then summed to a total score between 0 and 24. The SOFA score was recognized as a potential endpoint for randomized controlled trials (RCTs) when serially measured scores were found to be associated with mortality independent of admission score [2–4]. Due to its scalar nature, demonstrating a treatment effect on SOFA score requires a smaller sample size than demonstrating an effect on (dichotomous) mortality. This has led to increasing popularity of the SOFA score as a primary or secondary endpoint in RCTs.

The SOFA score is an intrinsically informative endpoint because it can be used to evaluate the effects of treatment on organ dysfunction, a primary focus of intensive care. However, it should be noted that a treatment that improves SOFA may not necessarily reduce mortality, or vice versa [5–8]. Mortality may be substantially influenced by factors that are not captured by the SOFA score. To extrapolate treatment effects from an intermediate outcome to a clinical outcome, effects of an intervention on the intermediate outcome (SOFA score) must reliably predict the overall effect on the clinical outcome (mortality) [6, 7].

The reliability of the SOFA score to predict mortality is complicated by the different derivatives of the SOFA score that are currently in use. Some authors report the SOFA score on one or more fixed days after randomization (Fixed-day SOFA). Others choose to report the Delta SOFA score, which is variably defined as the score on a fixed day after randomization minus the baseline score, or as the maximum score during the ICU stay minus the baseline score.

Reporting Fixed-day SOFA allows readers to compare mean organ dysfunction in the trial arms, while Delta SOFA allows readers to compare the trajectory of organ dysfunction from baseline in the trial arms. Other SOFA derivatives include the maximum score during the ICU stay, the mean score during the ICU stay or the score at the day of discharge or death. Neither Fixed-day SOFA nor Delta SOFA, or any of the other derivatives seem to be uniformly superior predictors for mortality in observational cohorts [4].

There are several unresolved issues around the validity of the SOFA score as an endpoint. First, the *responsiveness* of the SOFA score to intervention-induced change in mortality risk has not been quantified. It is unclear how the SOFA score changes in response to a treatment that changes the mortality risk within a specific timeframe. Second, the *consistency* of the SOFA score to reflect changes in underlying mortality risk has not been quantified. Even if true mortality-modifying treatments effects are reflected in the SOFA score on average, the validity of the SOFA score as an endpoint is doubtful if this relationship is inconsistent. Third, it is unclear which derivative of the SOFA score is the most appropriate endpoint.

Therefore, the aim of the present study was to quantify the *responsiveness* and the *consistency* of different SOFA derivatives to reflect treatment effects on mortality. The results from this study may aid clinical decision makers in the interpretation of trials that use SOFA as an endpoint, and may help investigators choose the most appropriate SOFA derivative in the design of future RCTs.

## Methods

### Overview

The protocol for this review was prospectively registered in Prospero (number CRD42016034014) [9]. Using a comprehensive search strategy, we sought to identify all published RCTs that reported both mortality and any SOFA derivative as an endpoint. For each RCT, we recorded between-group differences in mortality and between-group differences in the respective SOFA derivative. The data from all RCTs reporting a specific SOFA derivative were then aggregated using meta-regression. Responsiveness was quantified as the slope between the effects of treatment on mortality and on SOFA. Consistency was quantified using the meta-analytical parameter *I* ^*2*^.

### Inclusion, search strategy and recorded variables

Eligible for inclusion were RCTs in adult intensive care unit (ICU) patients reporting both a derivative of SOFA and a measure of mortality as primary or secondary endpoints. The search was limited to reports in English. The PubMed and Embase databases were queried using the term ‘(sofa OR "sepsis-related organ failure" OR "sepsis related organ failure" OR "sequential organ failure") AND (random* OR RCT)’. The query was last repeated on May 1, 2016.

For each RCT, we recorded and categorized the trial population, the type of intervention being tested, single-center or multicenter design and the primary endpoint. RCTs were graded according to the Jadad scale [10]. For each treatment group in each RCT, we recorded the sample size, baseline SOFA score, all reported serial SOFA scores and the reported mortality rates. Data extraction was independently performed by dual entry (HJdG and IG). Conflicting entries were resolved by consensus, with a final decision by a third author (HMO).

### Quantifying responsiveness and consistency

For each RCT, mortality was expressed as the odds ratio (OR) of treatment vs. control group mortality. For studies reporting multiple measures of mortality, one measure was chosen in the following order: mortality measure reported as the primary endpoint; 28-day mortality; hospital mortality; 90-day mortality; ICU mortality. For the SOFA score, we computed the standardized difference between the control and intervention groups, defined as the between-group difference in SOFA score divided by the standard deviation (SD) of the SOFA score (square root of the mean of variances of both groups). The standardized difference was used instead of the absolute difference to normalize the SOFA effect sizes across trials with different SOFA score distributions. When the SOFA score was reported as the median and IQR, the median was used as the best unbiased estimator of the mean and the SD was approximated as IQR/1.35. The SD of the SOFA score was imputed for six studies using the mean SD for the specific SOFA category.

A mixed-effects meta-regression model was used with log(OR) as the dependent variable, SOFA score (standardized difference) as the fixed effect independent variable and a random intercept for each study. The random intercept per study was applied to model heterogeneity explicitly. Fixed-effects and mixed-effects models produce identical results in the absence of significant between-study heterogeneity, but a mixed-effects model leads to appropriately increased standard errors when significant heterogeneity occurs. Each study was weighted by the inverse of the sampling variance of the mortality OR (a function of mortality rate and sample size). A restricted maximum likelihood estimator was used to estimate heterogeneity. Residuals were checked for normality and the goodness of fit of the log-linear model was compared to quadratic and power models.

The *responsiveness* of SOFA to mortality was measured by the coefficient that determines the slope between the standardized between-group difference in SOFA and the between-group mortality OR. The overall mortality effect explained by SOFA was quantified by the regression coefficient of determination (*R* ^2^).

The *consistency* of the relationship between SOFA and mortality was measured by *I* ^*2*^, which describes the percentage total variability that is unexplained by sampling error (chance) [11]. The cause of residual heterogeneity was explored by adding study-level explanatory variables (e.g. baseline SOFA and trial characteristics) as regressors in the model.

The meta-regression was performed for each derivative of the SOFA score. The different SOFA derivatives were categorized into Fixed-day SOFA (subcategorized into Early fixed-day SOFA and Late fixed-day SOFA), Delta SOFA (subcategorized into Delta fixed-day SOFA and Delta maximum SOFA), and other SOFA derivatives (Maximum SOFA, Mean SOFA and Discharge SOFA). RCTs recurred in multiple categories if more than one SOFA derivative was reported.

Pre-planned subgroup analyses were performed using RCTs in patients with sepsis (the SOFA score was originally designed to quantify sepsis-related organ failure), the 50% largest RCTs by sample size and the RCTs with a Jadad scale of 3 or higher (out of 5).

The year of publication, sample size and Jadad scale were compared between RCTs that reported different SOFA derivatives using analysis of variance (ANOVA). All reported *p* values were corrected for multiple comparisons using the Hommel method [12]. The regression analyses were performed in R using the metafor package [13]. The dataset generated and analyzed is available in Additional file 1.

## Results

### Characteristics of the included studies

**Fig. 1:**
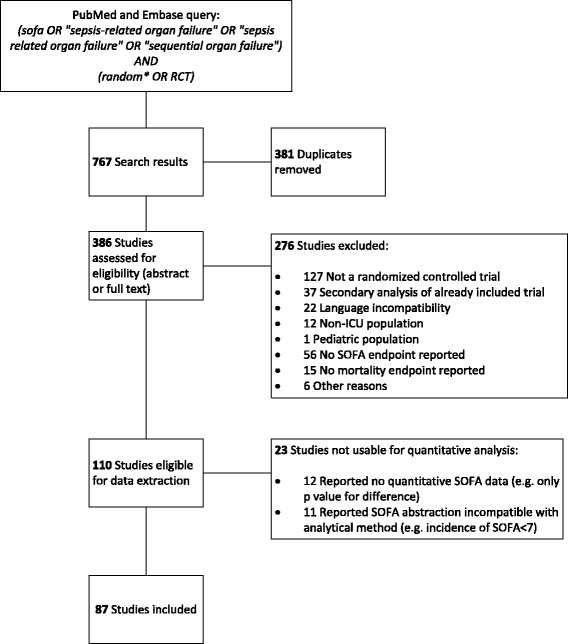
Flowchart of the search strategy and included trials. *SOFA* sequential organ failure assessment, *RCT* randomized controlled trial

**Fig. 2:**
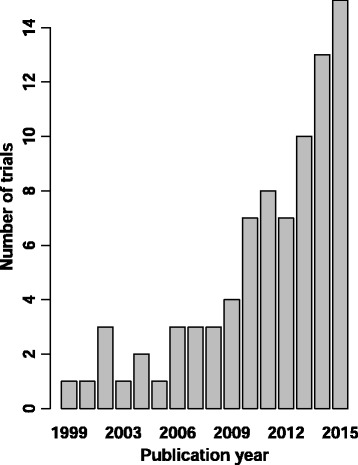
Included trials by publication year

The search and screening strategy identified 87 RCTs that were eligible and usable for quantitative analysis (Fig. 1). Characteristics of the included RCTs are summarized in Table 1. Most RCTs were performed in patients with severe sepsis or septic shock. The included RCTs were small to moderate in size with a median number of included patients of 64 patients (IQR 40–147). There were 18 RCTs (21%) that included more than 200 patients. Nineteen RCTs (22%) used SOFA as a primary endpoint and 68 (78%) reported SOFA as a secondary endpoint. Figure 2 shows the increasing use of the SOFA score as an endpoint over time.

**Table 1:** Characteristics of included trials

Characteristic	Number of trials (% of 87 included) or median (IQR)
Trial population, *n* (%)	
Severe sepsis or septic shock	35 (40%)
Mixed ICU population	24 (28%)
Specific organ dysfunction	13 (15%)
Trauma	4 (5%)
Cardiac surgery	2 (2%)
Other	9 (10%)
Trial intervention, *n* (%)	
Drug	47 (54%)
Treatment bundle	12 (14%)
Device	10 (11%)
Nutrition	8 (9%)
Ventilation-related	4 (5%)
Other	6 (7%)
Jadad scale, median (IQR)	3 (2 – 3)
Jadad scale ≤1, *n* (%)	14 (16%)
Multicenter design, *n* (%)	40 (46%)
Sample size per trial, median (IQR)	64 (40 – 147)
Mean or median baseline SOFA score, median (IQR)	8.5 (7 – 10)
Mortality rate, median (IQR)	28% (19% – 36%)
Primary endpoint, *n* (%)	
SOFA score	19 (22%)
Mortality	14 (16%)
Other	36 (41%)
Not specified	18 (21%)

*ICU* intensive care unit, *IQR* interquartile range, *SOFA* sequential organ failure assessment

The different SOFA derivatives that were used as endpoints in the included trials were sorted into the categories Fixed-day SOFA, Delta SOFA and other SOFA derivatives (Table 2). Fixed-day SOFA was subcategorized into Early fixed-day SOFA (score before day 7) and Late fixed-day SOFA (score on day 7 or later). Delta SOFA was subcategorized into Delta fixed-day SOFA and Delta maximum SOFA. Other SOFA derivatives were Maximum SOFA, Mean SOFA and Discharge SOFA. Mean SOFA and Discharge SOFA were used in only three RCTs and were therefore not analyzed for responsiveness and consistency. There were 46 RCTs (53%) that reported the effects of treatment on 28-day mortality, 17 (19%) that reported hospital mortality, 11 (13%) that reported long-term mortality and 13 (15%) that reported ICU mortality. The RCTs reporting different SOFA derivatives did not differ by year of publication (*p* = 0.616), sample size (*p* = 0.721), primary mortality measure (28-day vs. hospital vs. ICU) (*p* = 0.358) or Jadad scale (*p* = 0.976).

**Table 2:** SOFA derivatives used as endpoints

SOFA derivative	Description	Included RCTs
Fixed-day SOFA	SOFA score on a fixed day after randomization	58^a^
Early fixed-day SOFA	SOFA score on days 2, 3, 4 or 5 after randomization	55^a^
Late fixed-day SOFA	SOFA score on days 7, 10 or 14 after randomization	32^a^
Delta SOFA	Trajectory of SOFA score from baseline	25
Delta fixed-day SOFA	SOFA score on a fixed day after randomization minus baseline SOFA score	18
Delta maximum SOFA	Maximum SOFA score during ICU stay minus baseline SOFA score	7
Other SOFA derivatives		
Maximum SOFA	Maximum SOFA score during ICU stay	9
Mean SOFA	Mean SOFA score during ICU stay	3
Discharge SOFA	SOFA score at ICU discharge or death	3

^a^Twenty-nine trials reported both early and late SOFA scores. *SOFA* sequential organ failure assessment

### Relationship between SOFA and mortality endpoints

**Fig. 3:**
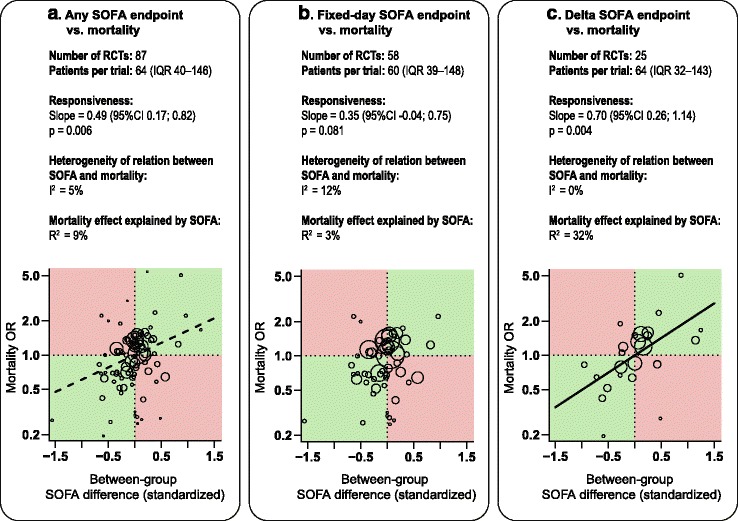
Regression analyses of the relationship between the RCT treatment effects on mortality vs. (**a**) any SOFA endpoint, (**b**) Fixed-day SOFA, and (**c**) Delta SOFA. The size of the circle is proportional to the RCT sample size. RCTs in the *green quadrants* show agreement between SOFA and the effects on mortality (e.g. lower SOFA and lower mortality), while RCTs in the *red quadrants* show conflicting effects (lower SOFA but higher mortality or vice versa). *Broken line* significant association with residual heterogeneity; *solid line* significant association without residual heterogeneity. SOFA sequential organ failure assessment, *RCT* randomized controlled trial, *OR* odds ratio

Figure 3 displays the meta-regression results of all included trials (*n* = 87) and the two most frequently used SOFA derivatives: Fixed-day SOFA (*n* = 58) and Delta SOFA (*n* = 25).

Among the 87 RCTs that used any SOFA derivative as an endpoint, there was significant responsiveness between the SOFA endpoint and mortality (slope = 0.49, *p* = 0.006, *I* ^2^ = 5%). Many RCTs reported conflicting treatment effects on SOFA vs. mortality (Fig. 3a, red quadrants). Overall, the *R* ^2^ statistic showed that 9% of the mortality effects were explained by SOFA.

For RCTs that used fixed-day SOFA (*n* = 58), there was no association between the SOFA endpoint and mortality (slope = 0.35, *p* = 0.08, *I* ^2^ = 12%) and the *R* ^2^ statistic showed that 3% of the mortality effects were explained by SOFA (Fig. 3b). The subcategories Early and Late fixed-day SOFA also displayed no significant association, with slope = 0.38, *p* = 0.261, *I* ^2^ = 14%, *R* ^2^ = 4% and slope = 0.18, *p* = 0.458, *I* ^2^ = 13%, R^2^ = 1%, respectively (Additional file 2: Appendix B, Figure B1).

RCTs that used Delta SOFA as an endpoint (*n* = 25) reported less conflicting results (Fig. 3c). Delta SOFA showed statistically significant responsiveness to mortality (slope = 0.70, *p* = 0.004, *I* ^2^ = 0%) and *R* ^2^ showed that 32% of the mortality effects were explained by SOFA (Fig. 3c). The subcategory Delta fixed-day SOFA (*n* = 18) showed similar results (slope = 0.74, *p* = 0.015, *I* ^2^ = 0%, *R* ^2^ = 35%). The subcategory Delta maximum SOFA (*n* = 7) showed non-significant responsiveness (slope = 0.54, *p* = 0.458, *I* ^2^ = 0%, *R* ^2^ = 9%) (Additional file 2: Appendix B, Figure B1). The heterogeneity of Delta SOFA was significantly lower than that of fixed-day SOFA (*p* < 0.001 using the *F* test on *tau*).

The only other SOFA derivative used by more than three RCTs was Maximum SOFA, which had the highest responsiveness estimate between SOFA and mortality. However, the relationship was not statistically significant, possibly because Maximum SOFA was used in only nine RCTs (slope = 1.03, *p* = 0.406, *I* ^2^ = 0%, *R* ^2^ = 36%) (Additional file 2: Appendix B, Figure B1).

### Subgroup analyses

We performed three subgroup analyses: (1) with RCTs in severe sepsis or septic shock populations; (2) with the largest 50% of RCTs by sample size; and (3) with RCTs scoring a Jadad quality scale of 3 or higher (out of a possible score of 5). No significant deviations from the main results were found for any of the subgroups. A notable result was that the responsiveness and consistency of fixed-day SOFA actually deteriorated when analyzing only high-quality RCTs and the largest RCTs. The results of the subgroup analyses can be found in Additional file 2: Appendix B, Table B2. Adding baseline between-group SOFA differences in the regression model did not improve the responsiveness or consistency of Fixed-day SOFA (slope 0.3, *p* = 0.34, *I* ^2^ = 17%, *R* ^2^ = 1%).

## Discussion

Our systematic review indicates that Delta SOFA score (but not Fixed-day SOFA score) reliably reflects between-group differences in mortality. Delta SOFA describes the change in organ function over time. It is strongly associated with mortality and explained 32% of the treatment effects on mortality. The subcategory Delta fixed-day SOFA performed similarly, but the subcategory Delta maximum SOFA was not significantly associated with mortality. Fixed-day SOFA, despite being the most frequently used derivative, was actually not associated with mortality and the estimated *R* ^2^ value was only 3%. The reason is that many RCTs using Fixed-day SOFA reported conflicting treatment effects on their SOFA score and mortality endpoints (i.e. a treatment that led to a better SOFA score but to worse mortality or vice versa). Maximum SOFA score had a high responsiveness estimate, but was possibly used in too few RCTs to be statistically significant. These results indicate that SOFA score obtained on a fixed day after randomization was not the most appropriate endpoint for RCTs and that Delta fixed-day SOFA performed best.

In small RCTs, conflicting results (opposing effects on SOFA and mortality) may be due to random chance alone. However, the employed meta-regression approach explicitly accounts for sampling variance. The presence of heterogeneity for Fixed-day SOFA therefore indicates that the effects on SOFA vs. mortality are more conflicted than would be expected by random chance alone. Similarly, the proportions of the mortality effects explained by the SOFA scores (the reported *R* ^2^ values) were weighted by study size to discount the effect of small outlier RCTs. In addition, the main findings were fundamentally unchanged when we analyzed only the largest and the highest-quality RCTs. In all, both the statistical methods and the subgroup analyses support the robustness of our findings.

The association between different SOFA derivatives and mortality has previously been evaluated in observational cohorts of critically ill patients [2–4]. We have focused on the treatment effects on SOFA scores and mortality in RCTs rather than on the observational association between SOFA and mortality. In observational studies, Fixed-day SOFA discriminated mortality risk with an area under the receiver operating characteristic (ROC) curve (AUC) of 0.73 to 0.85 [3, 14–16]. This moderate performance supports our finding that Fixed day SOFA is not robustly associated with mortality. The AUC for Maximum SOFA was 0.90 to 0.92 [3, 14, 17], which is in agreement with the relatively good performance of Maximum SOFA, although too few RCTs reported this SOFA derivative to draw robust conclusions. Delta fixed-day SOFA, which performed best in RCTs, has only been analyzed in a single observational cohort at day 2 and 3 (AUC of 0.76 and 0.62, respectively) [3].

The value of mortality as a gold standard endpoint for intensive care RCTs is the subject of longstanding debate [18, 19]. On the one hand, reducing mortality is a premier goal of intensive care treatment. In this light, an intermediate endpoint such as Delta SOFA score can be seen as a surrogate endpoint that needs to be validated against mortality [5, 6]. On the other hand, reducing morbidity (organ failure) in critically ill patients is intrinsically relevant. Mortality may be an insensitive endpoint because many of its determinants (such as older age or severe chronic illness) are not amenable to therapy. In this light, the SOFA score is a valuable endpoint in itself, and our finding that Delta SOFA explains 32% of the treatment effects on mortality further strengthens its relevance.

### Strengths and weaknesses of this study

The search strategy was designed to identify RCTs with a mention of randomization and SOFA score in the title, abstract or keywords. However, trials that used SOFA score as a secondary endpoint but did not mention SOFA in the abstract were possibly not identified. Twenty-two RCT reports not written in English were excluded, which may have compromised study power.

We used aggregated study-level data rather than individual patient data. This allowed us to use information from almost all available trials, thereby making the results generalizable across a broad spectrum of critical care RCTs. The results may have been influenced by publication bias if specific combinations of mortality and SOFA score effects are overrepresented or underrepresented. The included trials did not test similar interventions but rather represented a common biological pathway of multiple organ dysfunction as a determinant of ICU-related mortality. Statistical heterogeneity in the relationship between SOFA score and mortality therefore seemed inevitable, and we have modeled this explicitly by using mixed-model regression.

Using individual patient data from RCTs would have enabled different statistical methods that allow for a more precise estimate of the responsiveness between SOFA score and mortality [20, 21]. However, obtaining individual patient data from investigators would not have been a random and unbiased process, thereby compromising the generalizability of the results. Future research may be directed at individual patient data from one or several RCTs.

Among the analyzed RCTs, there was considerable heterogeneity in the reported mortality measures (e.g. 28-day, hospital or ICU) and the SOFA endpoints. Although we analyzed between-group differences rather than absolute mortality, this may have contributed to the unexpectedly poor performance of fixed-day SOFA. The reported SOFA endpoints were categorized to arrive at a statistically useful number of RCTs per SOFA derivative. Although our categorization broadly followed the naming conventions and classification of SOFA derivatives used elsewhere [3, 4], some dichotomies were arbitrary (e.g. the cutoff between early and late at 7 days).

It should also be stressed that the term Delta SOFA is defined differently throughout the literature: it is sometimes defined as the score on a fixed day minus the baseline score (delta fixed-day) or as the maximum score minus the baseline score (delta maximum). We found that on the whole, Delta SOFA was associated with mortality, but further analysis showed that this association was significant only for the RCTs that reported Delta fixed-day instead of Delta maximum SOFA.

An important limitation of this analysis lies in the sample size differences between SOFA derivatives. The statistical significance of the responsiveness (slope coefficient) depends on the magnitude of the coefficient, on the amount of residual heterogeneity and on the number of RCTs. Because the number of RCTs differs greatly between the different SOFA derivatives, the *p* values for the slope coefficients must be interpreted with caution. It should be noted, however, that fixed-day SOFA did not attain significance despite having a much larger number of RCTs than delta SOFA.

### Implications for the interpretation and design of clinical trials

The number of RCTs that use SOFA as an endpoint is increasing over time (Fig. 2). Yet the critical care community should be cautious about how much of a treatment effect on mortality can be extrapolated from a treatment effect on SOFA score. The reliability and validity of the SOFA score as an endpoint depends on several conditions: the appropriateness of the SOFA derivative; the adequacy of the sample size; the appropriateness of the timeframe; the correct scoring of discharged and deceased patients; and the validity of the individual SOFA components (especially the Glasgow coma score (GCS) in sedated patients).

First, for any RCT, the choice of SOFA derivative should be appropriate for the study design and research question. Based on the results from this review, Delta fixed-day SOFA reflects between-group mortality differences better than Fixed-day SOFA. Delta maximum SOFA and Maximum SOFA need further evaluation before their validity as an endpoint can be ascertained.

Second, given the mean reported standard deviation of Delta SOFA of 2.64, we can calculate the sample size requirements to detect a between-group difference in SOFA score with 80% power and 5% type-I error rate. With these parameters, 110 patients per group are required to detect a 1 point difference in Delta SOFA between the groups, which is associated with a mortality OR of 2 (*e* ^*1x*0.70^) (95% CI 1.3; 3.1). Detecting a 0.5 point difference in SOFA score (associated with a mortality OR of 1.4 (*e* ^*0.5x*0.70^) (95% CI 1.1; 1.8)) requires 440 patients per group. It should be noted that based on the included studies, a true between-group difference greater than 1 point in delta SOFA or a mortality OR greater than 2.0 seems unrealistic. Therefore, we suggest that RCTs using Delta SOFA as the primary endpoint should aim to detect a 1 point or smaller effect on SOFA (i.e. include no less than 110 patients per group.)

Third, the SOFA scores assigned to patients discharged from ICU and deceased patients should be carefully chosen and clearly described. Only a minority of the RCTs analyzed in this review described how these observations were registered. For any measurement point after ICU discharge, the SOFA score for that patient can be registered as the last observation carried forward or as 0, in the case of discharge, or 24 in the case of death. Simply assigning no score to discharged patients will obviously lead to bias that decreases the validity of the endpoint. Similarly, a plot showing the development of SOFA scores over time cannot be interpreted if discharged patients are not explicitly scored. Mean SOFA scores may paradoxically improve over time in one group because of greater early mortality, unless deceased patients are assigned a score of 24 or the last observation carried forward.

Fourth, since the mean time to reach the maximum SOFA score is different for each organ system, the timing of a fixed-day SOFA endpoint should be appropriate for the specific treatment target. For example, the effects on SOFA of a treatment that is primarily aimed at liver dysfunction should be assessed later than the effects on SOFA of a treatment that is primarily aimed at circulatory or respiratory dysfunction [2].

Fifth, the components of the SOFA score must be individually valid. The GCS is the most subjective variable in the SOFA score and its evaluation is often confounded by the use of sedatives. The interobserver agreement of the GCS ranges from moderate to very poor in validation studies of severity-of-illness scoring systems [22–24]. A modified SOFA score excluding the neurologic component could therefore be considered when the appropriate registration of the GCS has not been validated in the participating trial institutions or when it is found to be unreliable in the case of prolonged sedation.

Last and importantly, we recommend that investigators using SOFA as a primary endpoint should always report mortality as a secondary endpoint and should evaluate the within-trial association between SOFA and mortality using a proportion explained logistic regression analysis [20]. Reports of RCTs using a SOFA endpoint could then include a statement such as: “the treatment effect on the SOFA score explains 50% of the treatment effect on mortality, which supports the validity of this endpoint”, or, conversely: “the treatment effect on the SOFA score explains 10% of the treatment effect on mortality, which casts doubt on the predictive value of the SOFA endpoint in this trial”. This allows readers to better evaluate whether the effect of a treatment on SOFA is an accurate predictor of the effect of treatment on mortality in that specific trial.

## Conclusion

In this systematic analysis, 87 RCTs were included to evaluate the reliability of different SOFA derivatives to predict treatment effects on mortality. Based on study level data aggregated in this systematic review, Delta fixed-day SOFA appears to be most responsively and consistently associated with mortality. Fixed-day SOFA was the most frequently reported outcome measure among the reviewed RCTs but was not found to be associated with mortality. Maximum SOFA showed excellent responsiveness and consistency, but was used in too few trials for sufficient statistical power. We recommend that researchers planning to use SOFA as a trial endpoint should use Delta SOFA in preference to Fixed-day SOFA, choose an appropriate timeframe, describe how discharged and deceased patients are scored and evaluate the within-trial association between the SOFA endpoint and mortality.

## Additional files

Additional file 1: Generated and analyzed dataset (CSV 64 kb)

Additional file 2: Supplementary material (PDF 685 kb)

## Abbreviations

AUC: Area under the curve
GCS: Glasgow coma score
ICU: Intensive care unit
OR: Odds ratio
RCT: Randomized controlled trial
SOFA: Sequential organ failure assessment or Sepsis-related organ failure assessment
