# Supplementary material for: SOFA and mortality endpoints in randomized controlled trials: a systematic review and meta-regression analysis
Source: Crit Care. 2017 Feb 24;21:38. doi: 10.1186/s13054-017-1609-1 (PMC5324238; doi:10.1186/s13054-017-1609-1)
Supplement: Supplementary file 2 — Supplementary material (PDF 685 kb) [file 13054_2017_1609_MOESM2_ESM.pdf]

Supplementary material

Appendix A: List of included studies 2

Appendix B: Additional regression results 7

Appendix A: List of included studies

1. Acosta-Escribano, J. *et al.* Gastric versus transpyloric feeding in severe traumatic brain injury: a prospective, randomized trial. *Intensive Care Med.* **36,** 1532–9 (2010).
2. Agarwal, R., Srinivasan, A., Aggarwal, A. N. & Gupta, D. Adaptive support ventilation for complete ventilatory support in acute respiratory distress syndrome: a pilot, randomized controlled trial. *Respirology* **18,** 1108–15 (2013).
3. Al Tmimi, L. *et al.* Xenon anaesthesia for patients undergoing off-pump coronary artery bypass graft surgery: a prospective randomized controlled pilot trial. *Br. J. Anaesth.* **115,** 550–9 (2015).
4. Andrews, P. J. D. *et al.* Randomised trial of glutamine, selenium, or both, to supplement parenteral nutrition for critically ill patients. *BMJ* **342,** d1542 (2011).
5. Annborn, M. *et al.* The association of targeted temperature management at 33 and 36 °C with outcome in patients with moderate shock on admission after out-of-hospital cardiac arrest: a post hoc analysis of the Target Temperature Management trial. *Intensive Care Med.* **40,** 1210–9 (2014).
6. Barzegar, E., Ahmadi, A., Mousavi, S., Nouri, M. & Mojtahedzadeh, M. The Therapeutic Role of Vasopressin on Improving lactate Clearance During and After Vasogenic Shock: Microcirculation, Is It The Black Box? *Acta Med. Iran.* **54,** 15–23 (2016).
7. Beale, R. J. *et al.* Early enteral supplementation with key pharmaconutrients improves Sequential Organ Failure Assessment score in critically ill patients with sepsis: outcome of a randomized, controlled, double-blind trial. *Crit. Care Med.* **36,** 131–44 (2008).
8. Bein, T. *et al.* Continuous lateral rotational therapy and systemic inflammatory response in posttraumatic acute lung injury: results from a prospective randomised study. *Injury* **43,** 1892–7 (2012).
9. Berger, M. M. *et al.* Influence of early antioxidant supplements on clinical evolution and organ function in critically ill cardiac surgery, major trauma, and subarachnoid hemorrhage patients. *Crit. Care* **12,** R101 (2008).
10. Blackwood, B. *et al.* Tea tree oil (5%) body wash versus standard care (Johnson's Baby Softwash) to prevent colonization with methicillin-resistant Staphylococcus aureus in critically ill adults: a randomized controlled trial. *J. Antimicrob. Chemother.* **68,** 1193–9 (2013).
11. Briegel, J. *et al.* Stress doses of hydrocortisone reverse hyperdynamic septic shock: a prospective, randomized, double-blind, single-center study. *Crit. Care Med.* **27,** 723–732 (1999).
12. Brunkhorst, F. M. *et al.* Effect of empirical treatment with moxifloxacin and meropenem vs meropenem on sepsis-related organ dysfunction in patients with severe sepsis: a randomized trial. *JAMA* **307,** 2390–9 (2012).
13. Bulger, E. M. *et al.* A Novel Drug for Treatment of Necrotizing Soft-Tissue Infections: A Randomized Clinical Trial. *JAMA Surg.* **149,** 528–36 (2014).
14. Caliezi, C. *et al.* C1-inhibitor in patients with severe sepsis and septic shock: beneficial effect on renal dysfunction. *Crit. Care Med.* **30,** 1722–8 (2002).
15. Chu, L.-P., Zhou, J.-J., Yu, Y.-F., Huang, Y. & Dong, W.-X. Clinical effects of pulse high-volume hemofiltration on severe acute pancreatitis complicated with multiple organ dysfunction syndrome. *Ther. Apher. Dial.* **17,** 78–83 (2013).
16. Cicarelli, D. D., Benseñor, F. E. M. & Vieira, J. E. Effects of single dose of dexamethasone on patients with systemic inflammatory response. *São Paulo Med. J. = Rev. Paul. Med.* **124,** 90–5 (2006).
17. Cicarelli, D. D., Vieira, J. E. & Benseñor, F. E. M. Early dexamethasone treatment for septic shock patients: a prospective randomized clinical trial. *São Paulo Med. J. = Rev. Paul. Med.* **125,** 237–41 (2007).
18. Cornet, A. D. *et al.* Recombinant human activated protein C in the treatment of acute respiratory distress syndrome: a randomized clinical trial. *PLoS One* **9,** e90983 (2014).
19. Cruz, D. N. *et al.* Early use of polymyxin B hemoperfusion in abdominal septic shock: the EUPHAS randomized controlled trial. *JAMA* **301,** 2445–52 (2009).

20. Darenberg, J. *et al.* Intravenous immunoglobulin G therapy in streptococcal toxic shock syndrome: a European randomized, double-blind, placebo-controlled trial. *Clin. Infect. Dis.* **37,** 333–40 (2003).
21. de Azevedo, R. P., Freitas, F. G. R., Ferreira, E. M., Pontes de Azevedo, L. C. & Machado, F. R. Daily laxative therapy reduces organ dysfunction in mechanically ventilated patients: a phase II randomized controlled trial. *Crit. Care* **19,** 329 (2015).
22. Dubin, A. *et al.* Comparison of 6% hydroxyethyl starch 130/0.4 and saline solution for resuscitation of the microcirculation during the early goal-directed therapy of septic patients. *J. Crit. Care* **25,** 659.e1–8 (2010).
23. Dubois, M.-J. *et al.* Albumin administration improves organ function in critically ill hypoalbuminemic patients: A prospective, randomized, controlled, pilot study. *Crit. Care Med.* **34,** 2536–40 (2006).
24. Erb, J. *et al.* Influence of levosimendan on organ dysfunction in patients with severely reduced left ventricular function undergoing cardiac surgery. *J. Int. Med. Res.* **42,** 750–64 (2014).
25. Eslami, K. *et al.* Positive effect of septimeb on mortality rate in severe sepsis: a novel non antibiotic strategy. **20,** 20–40 (2012).
26. Fagon, J. Y. *et al.* Invasive and noninvasive strategies for management of suspected ventilator-associated pneumonia. A randomized trial. *Ann. Intern. Med.* **132,** 621–30 (2000).
27. Fowler, A. a *et al.* Phase I safety trial of intravenous ascorbic acid in patients with severe sepsis. *J. Transl. Med.* **12,** 32 (2014).
28. Gando, S. *et al.* A randomized, controlled, multicenter trial of the effects of antithrombin on disseminated intravascular coagulation in patients with sepsis. *Crit. Care* **17,** R297 (2013).
29. Grau-Carmona, T. *et al.* Effect of an enteral diet enriched with eicosapentaenoic acid, gamma-linolenic acid and anti-oxidants on the outcome of mechanically ventilated, critically ill, septic patients. *Clin. Nutr.* **30,** 578–84 (2011).
30. Guidet, B. *et al.* Assessment of hemodynamic efficacy and safety of 6% hydroxyethylstarch 130/0.4 vs. 0.9% NaCl fluid replacement in patients with severe sepsis: the CRYSTMAS study. *Crit. Care* **16,** R94 (2012).
31. Hall, T. C. *et al.* A randomized controlled trial investigating the effects of parenteral fish oil on survival outcomes in critically ill patients with sepsis: a pilot study. *JPEN. J. Parenter. Enteral Nutr.* **39,** 301–12 (2015).
32. Hosny, M., Nahas, R., Ali, S., Elshafei, S. A. & Khaled, H. Impact of oral omega-3 fatty acids supplementation in early sepsis on clinical outcome and immunomodulation. *Egypt. J. Crit. Care Med.* **1,** 119–126 (2013).
33. Huang, Z., Wang, S.-R., Su, W. & Liu, J.-Y. Removal of humoral mediators and the effect on the survival of septic patients by hemoperfusion with neutral microporous resin column. *Ther. Apher. Dial.* **14,** 596–602 (2010).
34. Iapichino, G. *et al.* Tight glycemic control does not affect asymmetric-dimethylarginine in septic patients. *Intensive Care Med.* **34,** 1843–50 (2008).
35. Jabre, P. *et al.* Etomidate versus ketamine for rapid sequence intubation in acutely ill patients: a multicentre randomised controlled trial. *Lancet (London, England)* **374,** 293–300 (2009).
36. Komisarof, J. A. *et al.* N-acetylcysteine for patients with prolonged hypotension as prophylaxis for acute renal failure (NEPHRON). *Crit. Care Med.* **35,** 435–41 (2007).
37. Kruger, P. *et al.* A multicenter randomized trial of atorvastatin therapy in intensive care patients with severe sepsis. *Am. J. Respir. Crit. Care Med.* **187,** 743–50 (2013).
38. Larsen, F. S. *et al.* High-volume plasma exchange in patients with acute liver failure: An open randomised controlled trial. *J. Hepatol.* **64,** 69–78 (2016).
39. Lasocki, S., Labat, F., Plantefeve, G., Desmard, M. & Mentec, H. A long-term clinical evaluation of autoflow during assist-controlled ventilation: a randomized controlled trial. *Anesth. Analg.* **111,** 915–21 (2010).
40. Lauzier, F., Lévy, B., Lamarre, P. & Lesur, O. Vasopressin or norepinephrine in early hyperdynamic septic shock: a randomized clinical trial. *Intensive Care Med.* **32,** 1782–9 (2006).

41. Lemiale, V. *et al.* Effect of Noninvasive Ventilation vs Oxygen Therapy on Mortality Among Immunocompromised Patients With Acute Respiratory Failure: A Randomized Clinical Trial. *JAMA* **314,** 1711–9 (2015).
42. Leone, M. *et al.* De-escalation versus continuation of empirical antimicrobial treatment in severe sepsis: a multicenter non-blinded randomized noninferiority trial. *Intensive Care Med.* **40,** 1399–408 (2014).
43. Li, M.-Q. *et al.* Effect of the Shenfu Injection Combined with Early Goal-Directed Therapy on Organ Functions and Outcomes of Septic Shock Patients. *Cell Biochem. Biophys.* (2015). doi:10.1007/s12013-015-0537-4
44. Lu, N.-F. *et al.* Improved sepsis bundles in the treatment of septic shock: a prospective clinical study. *Am. J. Emerg. Med.* **33,** 1045–9 (2015).
45. Mahmoodpoor, A. *et al.* Examination of Setarud (IMOD™) in the management of patients with severe sepsis. *Daru* **18,** 23–8 (2010).
46. Manzanares, W. *et al.* High-dose selenium for critically ill patients with systemic inflammation: pharmacokinetics and pharmacodynamics of selenious acid: a pilot study. *Nutrition* **26,** 634–40 (2010).
47. Manzanares, W. *et al.* High-dose selenium reduces ventilator-associated pneumonia and illness severity in critically ill patients with systemic inflammation. *Intensive Care Med.* **37,** 1120–7 (2011).
48. Mishra, V. *et al.* Effect of selenium supplementation on biochemical markers and outcome in critically ill patients. *Clin. Nutr.* **26,** 41–50 (2007).
49. Moreno, R. *et al.* Time course of organ failure in patients with septic shock treated with hydrocortisone: results of the Corticus study. *Intensive Care Med.* **37,** 1765–72 (2011).
50. Morrison, L. J. *et al.* The Toronto prehospital hypertonic resuscitation--head injury and multiorgan dysfunction trial: feasibility study of a randomized controlled trial. *J. Crit. Care* **26,** 363–72 (2011).
51. Mouncey, P. R. *et al.* Trial of early, goal-directed resuscitation for septic shock. *N. Engl. J. Med.* **372,** 1301–11 (2015).
52. Müller, M. C. *et al.* Transfusion of fresh-frozen plasma in critically ill patients with a coagulopathy before invasive procedures: a randomized clinical trial (CME). *Transfusion* **55,** 26–35; quiz 25 (2015).
53. Najafi, A. *et al.* The Comparison of Procalcitonin Guidance Administer Antibiotics with Empiric Antibiotic Therapy in Critically Ill Patients Admitted in Intensive Care Unit. *Acta Med. Iran.* **53,** 562–7 (2015).
54. Najafi, A. *et al.* The immunological benefit of higher dose N-acetyl cysteine following mechanical ventilation in critically ill patients. *Daru* **22,** 57 (2014).
55. Oppert, M. *et al.* Low-dose hydrocortisone improves shock reversal and reduces cytokine levels in early hyperdynamic septic shock. *Crit. Care Med.* **33,** 2457–64 (2005).
56. Papazian, L. *et al.* Effect of statin therapy on mortality in patients with ventilator-associated pneumonia: a randomized clinical trial. *JAMA* **310,** 1692–700 (2013).
57. Pavlovic, G. *et al.* Impact of early haemodynamic goal-directed therapy in patients undergoing emergency surgery: an open prospective, randomised trial. *J. Clin. Monit. Comput.* **30,** 87–99 (2016).
58. Payen, D. M. *et al.* Early use of polymyxin B hemoperfusion in patients with septic shock due to peritonitis: a multicenter randomized control trial. *Intensive Care Med.* **41,** 975–84 (2015).
59. Payen, J.-F. *et al.* Corticosteroid after etomidate in critically ill patients: a randomized controlled trial. *Crit. Care Med.* **40,** 29–35 (2012).
60. Perner, A. *et al.* Hydroxyethyl starch 130/0.42 versus Ringer's acetate in severe sepsis. *N. Engl. J. Med.* **367,** 124–34 (2012).
61. Petros, S., Horbach, M., Seidel, F. & Weidhase, L. Hypocaloric vs Normocaloric Nutrition in Critically Ill Patients: A Prospective Randomized Pilot Trial. *JPEN. J. Parenter. Enteral Nutr.* **40,** 242–9 (2016).
62. Pettilä, V. *et al.* APCAP--activated protein C in acute pancreatitis: a double-blind randomized human pilot trial. *Crit. Care* **14,** R139 (2010).
63. Ralph, C. J., Tanser, S. J., Macnaughton, P. D. & Sinclair, D. G. A randomised controlled trial investigating the effects of dopexamine on gastrointestinal function and organ dysfunction in the critically ill. *Intensive Care Med.* **28,** 884–90 (2002).

64. Reinhart, K. *et al.* Open randomized phase II trial of an extracorporeal endotoxin adsorber in suspected Gram-negative sepsis. *Crit. Care Med.* **32,** 1662–8 (2004).
65. Richir, M. C. *et al.* The effect of rosiglitazone on asymmetric dimethylarginine (ADMA) in critically ill patients. *Pharmacol. Res.* **60,** 519–24 (2009).
66. Rijnders, B. J., Peetermans, W. E., Verwaest, C., Wilmer, A. & Van Wijngaerden, E. Watchful waiting versus immediate catheter removal in ICU patients with suspected catheter-related infection: a randomized trial. *Intensive Care Med.* **30,** 1073–80 (2004).
67. Rixen, D. *et al.* Randomized, controlled, two-arm, interventional, multicenter study on risk-adapted damage control orthopedic surgery of femur shaft fractures in multiple-trauma patients. *Trials* **17,** 47 (2016).
68. Savioli, M. *et al.* Tight glycemic control may favor fibrinolysis in patients with sepsis. *Crit. Care Med.* **37,** 424–31 (2009).
69. Scheeren, T. W. L., Wiesenack, C., Gerlach, H. & Marx, G. Goal-directed intraoperative fluid therapy guided by stroke volume and its variation in high-risk surgical patients: a prospective randomized multicentre study. *J. Clin. Monit. Comput.* **27,** 225–33 (2013).
70. Schortgen, F. *et al.* Fever control using external cooling in septic shock: a randomized controlled trial. *Am. J. Respir. Crit. Care Med.* **185,** 1088–95 (2012).
71. Sehgal, I. S., Agarwal, R., Aggarwal, A. N. & Jindal, S. K. A randomized trial of Mycobacterium w in severe sepsis. *J. Crit. Care* **30,** 85–9 (2015).
72. Shirai, K., Yoshida, S., Matsumaru, N., Toyoda, I. & Ogura, S. Effect of enteral diet enriched with eicosapentaenoic acid, gamma-linolenic acid, and antioxidants in patients with sepsis-induced acute respiratory distress syndrome. *J. intensive care* **3,** 24 (2015).
73. Shum, H. P., Leung, Y. W., Lam, S. M., Chan, K. C. & Yan, W. W. Alteco endotoxin hemoadsorption in Gram-negative septic shock patients. *Indian J. Crit. Care Med.* **18,** 783–8 (2014).
74. Tan, M., Zhu, J.-C., Du, J., Zhang, L.-M. & Yin, H.-H. Effects of probiotics on serum levels of Th1/Th2 cytokine and clinical outcomes in severe traumatic brain-injured patients: a prospective randomized pilot study. *Crit. Care* **15,** R290 (2011).
75. Tokarik, M., Sjöberg, F., Balik, M., Pafcuga, I. & Broz, L. Fluid therapy LiDCO controlled trial-optimization of volume resuscitation of extensively burned patients through noninvasive continuous real-time hemodynamic monitoring LiDCO. *J. Burn Care Res.* **34,** 537–42
76. Trzeciak, S. *et al.* Randomized controlled trial of inhaled nitric oxide for the treatment of microcirculatory dysfunction in patients with sepsis*. *Crit. Care Med.* **42,** 2482–92 (2014).
77. Tugrul, S. *et al.* The effects of IgM-enriched immunoglobulin preparations in patients with severe sepsis [ISRCTN28863830]. *Crit. Care* **6,** 357–62 (2002).
78. Valenta, J., Brodska, H., Drabek, T., Hendl, J. & Kazda, A. High-dose selenium substitution in sepsis: a prospective randomized clinical trial. *Intensive Care Med.* **37,** 808–15 (2011).
79. van der Voort, P. H. J. *et al.* Testing a conceptual model on early opening of the microcirculation in severe sepsis and septic shock: a randomised controlled pilot study. *Eur. J. Anaesthesiol.* **32,** 189–98 (2015).
80. van Zanten, A. R. H. et al. High-protein enteral nutrition enriched with immune-modulating nutrients vs standard high-protein enteral nutrition and nosocomial infections in the ICU: a randomized clinical trial. JAMA 312, 514–24 (2014).
81. Wald, R. *et al.* Optimal Mode of clearance in critically ill patients with Acute Kidney Injury (OMAKI)--a pilot randomized controlled trial of hemofiltration versus hemodialysis: a Canadian Critical Care Trials Group project. *Crit. Care* **16,** R205 (2012).
82. Walsh, T. S. *et al.* Restrictive versus liberal transfusion strategies for older mechanically ventilated critically ill patients: a randomized pilot trial. *Crit. Care Med.* **41,** 2354–63 (2013).
83. Wang, C.-H. *et al.* Adjuvant treatment with a mammalian target of rapamycin inhibitor, sirolimus, and steroids improves outcomes in patients with severe H1N1 pneumonia and acute respiratory failure. *Crit. Care Med.* **42,** 313–21 (2014).
84. Wernerman, J. *et al.* Scandinavian glutamine trial: a pragmatic multi-centre randomised clinical trial of intensive care unit patients. *Acta Anaesthesiol. Scand.* **55,** 812–8 (2011).
85. Wu, J. *et al.* The efficacy of thymosin alpha 1 for severe sepsis (ETASS): a multicenter, single-blind, randomized and controlled trial. *Crit. Care* **17,** R8 (2013).

86. Zhang, Z., Ni, H. & Qian, Z. Effectiveness of treatment based on PiCCO parameters in critically ill patients with septic shock and/or acute respiratory distress syndrome: a randomized controlled trial. *Intensive Care Med.* **41,** 444–51 (2015).
87. Zu, H., Li, Q., Huang, P. & Wang, X. Therapeutic Value of Blood Purification and Prognostic Utilities of Early Serum Procalcitonin, C Reactive Protein, and Brain Natriuretic Peptide Levels in Severely Burned Patients with Sepsis. *Cell Biochem. Biophys.* **17,** 306 (2015).

Appendix B: Additional regression results

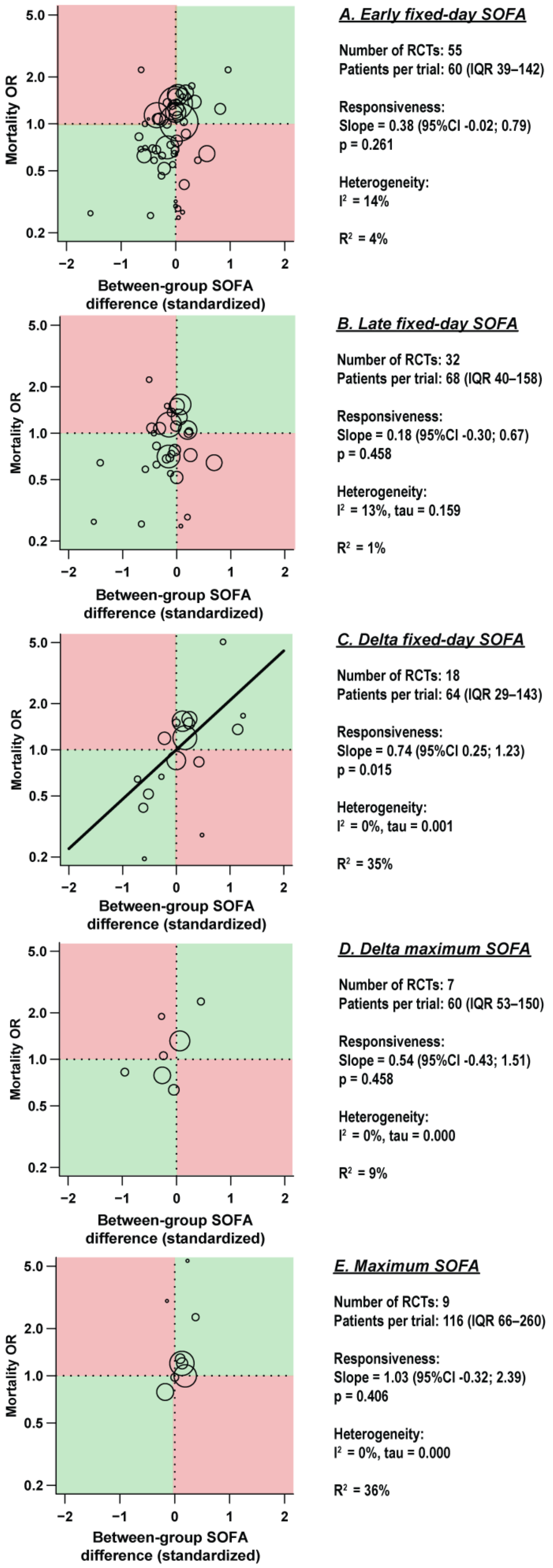

Figure B-1. Meta-regression analyses of the relation between RCT treatment effects on SOFA vs. mortality. RCTs in the green quadrants show agreement between SOFA and mortality effects (e.g. lower SOFA *and* lower mortality), while RCTs in the red quadrants show conflicting effects (lower SOFA but higher mortality or vice versa).

SOFA, Sequential Organ Failure Assessment; RCT, Randomized Controlled trial; OR, odds ratio.

Table B-1. Results of subgroup analyses

SOFA derivative	No. of RCTs	Slope coefficient (95% CI)	Slope p-value	I^2 (hetero-geneity)	R^2
**All RCTs (as reported in results section):**					
Fixed-day SOFA	58	0.35 (-0.04; 0.75)	0.081	12%	3%
Early fixed-day SOFA	55	0.38 (-0.02; 0.79)	0.261	14%	4%
Late fixed-day SOFA	32	0.18 (-0.30; 0.67)	0.458	13%	1%
Delta SOFA	25	0.70 (0.26; 1.14)	0.004	0%	32%
Delta fixed-day SOFA	18	0.74 (0.25; 1.23)	0.015	0%	35%
Delta maximum SOFA	7	0.54 (-0.43; 1.51)	0.458	0%	9%
Maximum SOFA	9	1.03 (-0.32; 2.39)	0.406	0%	36%
**Only RCTs with sepsis population:**					
Fixed-day SOFA	22	0.45 (-0.21; 1.12)	0.512	22%	2%
Early fixed-day SOFA	21	0.44 (-0.22; 1.11)	0.512	23%	1%
Late fixed-day SOFA	11	0.82 (-0.72; 2.35)	0.594	24%	1%
Delta SOFA	14	0.77 (0.15; 1.40)	0.091	2%	28%
Delta fixed-day SOFA	11	1.01 (0.23; 1.78)	0.064	5%	36%
Delta maximum SOFA	3	0.29 (-0.78; 1.35)	0.597	0%	0%
Maximum SOFA	2	1.47 (-1.56; 4.52)	0.597	0%	90%
**Only the 50% of RCTs with the largest sample sizes:**					
Fixed-day SOFA	28	0.15 (-0.36; 0.66)	0.564	23%	1%
Early fixed-day SOFA	26	0.19 (-0.34; 0.73)	0.564	26%	1%
Late fixed-day SOFA	17	-0.34 (-1.05; 0.37)	0.564	20%	0%
Delta SOFA	13	0.71 (0.16; 1.25)	0.076	0%	34%
Delta fixed-day SOFA	10	0.66 (0.09; 1.23)	0.131	0%	33%
Delta maximum SOFA	3	1.26 (-0.76; 3.29)	0.564	0%	23%
Maximum SOFA	6	0.77 (-0.71; 2.25)	0.564	0%	43%
**Only RCTs with a Jadad scale of 3 or more (out of 5):**					
Fixed-day SOFA	34	0.22 (-0.36; 0.84)	0.668	21%	0%
Early fixed-day SOFA	33	0.23 (-0.41; 0.87)	0.668	24%	0%
Late fixed-day SOFA	18	-0.16 (-0.91; 0.58)	0.668	21%	1%
Delta SOFA	17	0.78 (0.26; 1.29)	0.023	0%	36%
Delta fixed-day SOFA	11	0.74 (0.19; 1.29)	0.049	0%	37%
Delta maximum SOFA	6	1.02 (-0.49; 2.55)	0.644	0%	19%
Maximum SOFA	9	1.03 (-0.32; 2.39)	0.542	0%	36%

SOFA, Sequential Organ Failure Assessment; RCT, Randomized Controlled Trial.
